# Rural-urban differentials in the association between sex preference for children and marital dissolution in sub-Saharan Africa

**DOI:** 10.1371/journal.pone.0291435

**Published:** 2023-10-05

**Authors:** Joshua Okyere, Eugene Budu, Bright Opoku Ahinkorah, Richard Gyan Aboagye, Abdul-Aziz Seidu, Sanni Yaya

**Affiliations:** 1 Department of Population and Health, University of Cape Coast, Cape Coast, Ghana; 2 Department of Nursing, College of Health Sciences, Kwame Nkrumah University of Science and Technology, Kumasi, Ghana; 3 Korle Bu Teaching Hospital, Accra, Ghana; 4 School of Public Health, Faculty of Health, University of Technology Sydney, Sydney, Australia; 5 REMS Consult Limited, Sekondi-Takoradi, Western Region, Ghana; 6 Department of Family and Community Health, Fred N. Binka School of Public Health, University of Health and Allied Sciences, Hohoe, Ghana; 7 Department of Estate Management, Takoradi Technical University, Takoradi, Ghana; 8 College of Public Health, Medical and Veterinary Sciences, James Cook University, Douglas, Australia; 9 School of International Development and Global Studies, University of Ottawa, Ottawa, Canada; 10 The George Institute for Global Health, Imperial College London, London, United Kingdom; Adam Mickiewicz University Faculty of Biology: Uniwersytet im Adama Mickiewicza w Poznaniu Wydzial Biologii, POLAND

## Abstract

**Background:**

Marital dissolution, which refers to being divorced or separated, is considered one of the most dramatic demographic events that significantly disrupt families. Unearthing the factors predicting marital dissolution would support actions to reduce the incidence of this phenomenon. The present study sought to examine the association between sex preference for children and marital dissolution segregated by place of residence.

**Methods:**

Data for the study were extracted from the most recent Demographic and Health Surveys (DHS) of 25 countries in sub-Saharan Africa. Percentages were used to summarise the proportion of marital dissolution among women in sub-Saharan Africa. Binary logistic regression models were fitted to examine the association between sex preference for children and marital dissolution per place of residence. Results of the regression analysis were presented using adjusted odds ratios (aOR) with their respective 95% confidence interval (CI).

**Results:**

The overall prevalence of marital dissolution was 5.92% (95% CI: 5.83–6.00), and this ranged from 1.63% (95% CI: 1.41–1.85) in Burkina Faso to 15.62% (95% CI: 14.70–16.54) in Mozambique. In urban sub-Saharan Africa, the overall prevalence of marital dissolution was 8.88% (95% CI:8.78–8.99), with the lowest prevalence in Mali (3.30%; 95% CI: 2.91–3.69) and the highest in Uganda (18.60%; 95% CI: 17.95–19.25). For rural sub-Saharan Africa, the pooled prevalence was 4.11% (95% CI: 4.03–4.18), with the lowest (0.80%; 95% CI: 0.65–0.95) and highest (14.40%; 95% CI: 13.51–15.29) prevalences in Burkina Faso and Mozambique, respectively. Compared to women with no sex preference, the preference for boys was less likely to result in marital dissolution (aOR = 0.87; 95%CI = 0.83–0.90) in both urban and rural areas, whereas the preference for girls was more likely to result in marital dissolution (aOR = 1.06; 95%CI = 1.02–1.10). When the results were disaggregated by place of residence, in both urban (aOR = 0.87; 95%CI = 0.80–0.95) and rural areas (aOR = 0.87; 95%CI = 0.82–0.92), women who preferred boys were less likely to experience marital dissolution compared to those who had no preference. However, the preference for girls showed no statistically significant association with marital dissolution.

**Conclusion:**

Our study has shown that sex preference for children has a significant association with marital dissolution in both rural and urban areas in sub-Saharan Africa. Whereas the preference for male children serves as a protective factor against marital dissolution, the preferences for females was found to increase the likelihood of marital dissolution. Thus, underscoring a need for anti-marital dissolution campaigns and initiatives to prioritise the sensitisation of society about the value of female children. Religious groups and leaders can leverage their platform to quell sex preferences and dissuade marital dissolution. Policies and programmes aimed at reducing the risk of marital dissolution in sub-Saharan Africa must also focus on enlightening the population on intimate partner violence prevention.

## Background

Marital dissolution, which refers to being divorced or separated, is considered one of the most dramatic demographic events that significantly disrupt families [1]. This phenomenon is increasing across the globe. In Italy, the number of divorce cases increased from 82,469 in 2015 to 99,071 in 2016 [2]. Evidence also shows that the prevalence of divorce in the United States, Europe, and Norway was 46.4%, 45.5%, and 44.2%, respectively in 2018 [3, 4]. In Ethiopia, divorce increased from 24% in 2013 to 49.7% in 2015 [5], while in Nigeria, the prevalence of marital dissolution was estimated at 10.3% in 2018 [6].

The consequences of marital dissolution have been widely documented. Individuals who have been divorced report adverse psychosocial outcomes including depressive symptoms and loneliness [7, 8]. Some economic consequences have been found to be associated with marital dissolution [9]. Given these adverse effects of marital dissolution, prior studies have aimed to investigate the determinants of marital dissolution to develop interventions and programmes to reduce its incidence and mitigate any adverse effects that may be associated with divorce and separation. Broadly, demographic, attitudinal and cultural, economic, and legal factors have been identified to predict the risk of marital dissolution [1, 10, 11]. Demographic factors associated with the risk of marital dissolution include age, educational attainment, place of residence, and employment status [12, 13]. Infidelity, third-party intrusion, and lack of intimacy constitute some of the attitudinal and cultural risk factors of marital dissolution [10]. Also, the liberalisation of divorce laws contributes to the risk of marital dissolution [1]. Available evidence shows that wealth status, women’s participation in labour and women’s control over economic resources contribute substantially to marital dissolution [5]. However, there is growing interest in how child-related dynamics influence marital dissolution [6, 14].

According to Xu [14], children could contribute to a stronger marriage by fostering spousal interdependence and parental responsibility, thereby serving as a deterrent to marital dissolution. However, evidence suggests that the number of children is not the most significant factor that contributes to marital dissolution [15]; rather, it is the sex preferences of the child. Theoretically, the assumption that sex preferences contribute significantly to marital status is situated in the ‘father involvement hypothesis’ [16]. According to this theory, families with male-child preferences are less likely to experience marital dissolution because men are more involved in socialising male children than female children [16]. Consequently, it strengthens and stabilises marriages by fostering interdependence between partners. Also, in societies and cultures where male children are preferred, it is assumed that the child is of higher marital value compared to female children. Hence, having male children could further consolidate the women’s position in the husband’s family, making marital dissolution difficult to occur [14]. It is also important to note that such culturally contextualised sex preferences are most prevalent in rural areas [6]. Hence, there is a need to understand the rural-urban differences in sex preferences.

Despite the theoretical potential for sex preference for children to predict marital dissolution, there is limited empirical evidence in sub-Saharan Africa (SSA) to support the assertion. To the best of our knowledge, only one study has been conducted within the subregion to examine the influence of place of residence on sex preference for children as a predictor of marital dissolution [6]. Ajaero and Odimegwu’s [6] study was, however, limited to only Nigeria. Hence, it does not present the subregional status quo. The present study, therefore, narrows this knowledge gap by examining the association between sex preference for children and marital dissolution across the dimension of place of residence in 25 sub-Saharan African countries.

## Methods

### Data source

The most recent Demographic and Health Surveys (DHS) of 25 countries in SSA were used as the source of data. The DHS is a nationally representative survey conducted in over 85 low-and-middle-income countries worldwide [17]. DHS employs a descriptive cross-sectional design and samples respondents using a two-stage cluster sampling method. Detailed sampling technique has been highlighted in the literature [18]. Standardized structured questionnaires were used to collect data from the respondents on health indicators and demographic issues such as sex preference for children and marital status. We included a total of 275,031 women in this study (Table 1). Only women who had ever been married were included in the survey. The dataset used is freely available at https://dhsprogram.com/data/available-datasets.cfm. This paper was drafted with reference to the Strengthening the Reporting of Observational Studies in Epidemiology (STROBE) statement guidelines [19].

**Table 1:** Sample size per country.

Country	Survey year	Weighted Frequency	Weighted Percentage
Angola	2015–16	10550	3.84
Burkina Faso	2010	12861	4.68
Benin	2017–18	11891	4.32
Burundi	2016–17	12870	4.68
DR Congo	2013–14	13696	4.98
Cote d’Ivorie	2011–12	7412	2.70
Cameroon	2018	11156	4.05
Ethiopia	2016	11959	4.34
Gabon	2012	5970	2.17
Gambia	2019–20	8363	3.04
Kenya	2014	22759	8.28
Comoros	2012	3872	1.41
Liberia	2019	5456	1.98
Mali	2018	7973	2.90
Malawi	2015–16	18474	6.72
Mozambique	2011	5995	2.18
Nigeria	2018	31321	11.39
Namibia	2013	5924	2.15
Rwanda	2019–20	10196	3.71
Sierra Leone	2019	11519	4.19
Chad	2014–15	13103	4.76
Togo	2013–14	6971	2.53
Uganda	2016	13762	5.00
Zambia	2018	6278	2.54
Zimbabwe	2015	4016	1.46
Total		275031	100.00

### Outcome variable

The outcome variable for this study is marital dissolution, which was coded as either yes (1) or no (0). Since no direct question on family dissolution is contained in the DHS datasets, the variable of current marital status by women was used to generate the marital dissolution variable. Based on earlier studies [6, 11], women whose current marital status was divorced or separated were classified as being currently in marital dissolution and those who were married or widowed at the time of the survey were considered as those who have not experienced marital dissolution. Women who had never been married were excluded from the study sample.

### Explanatory variables

The key explanatory variable for the study is sex preference. To assess sex preference, we used two variables in the DHS that asked about (i) ideal number of boys, and (ii) ideal number of girls needed by couples [6]. Respondents whose ideal number of boys was equal to the ideal number of girls were categorized as those with “no sex preference”. Those whose ideal number of boys was more than their ideal number of girls were categorized as those with “male sex preference”, while those whose number of girls were more than their ideal number of boys were classified as having “female sex preference” [6]. Other explanatory variables (covariates) that were included in the study were age, educational level, employment status, religion, and intimate partner violence (individual level covariates). The rest were wealth index, place of residence and sub-region (contextual level variables). The explanatory variables considered in this study were selected based on their association with marital dissolution from the literature [6, 11, 20].

### Statistical analyses

Data for the study were analysed using Stata version 16.0. The data were weighted for under sampling and oversampling errors as per the survey design using the Stata ‘svyset’ command before data analyses. All the analyses were based on rural/urban place of residence. The analysis was carried out in four steps. First, a descriptive analysis was conducted to determine sample characteristics according to urban-rural residence (Table 2). Later, we used forest plots to show the prevalence of marital dissolution across the 25 countries included in the study using pe rcentages with 95% confidence interval (CI). Thereafter, we adopted a descriptive analysis to examine the prevalence of marriage dissolution by place of residence across the explanatory variables (Table 3). We used multilevel binary logistic regression analysis to examine the association between sex preference for children and marital dissolution, controlling for the covariates at the pooled level (combined rural and urban) (Table 4). To examine the association between sex preference for children and marital dissolution by place of residence, we used a multivariable binary logistic regression analysis (Table 5). The regression results were presented using crude odds ratio (cOR) and adjusted odds ratio (aOR) with their respective 95% CI. Statistical significance was set at p<0.05.

**Table 2:** Background characteristics of women in sub-Saharan Africa used in the study.

Variables	Urban		Rural	
	Weighted sample	Percentage	Weighted sample	Percentage
**Gender preference**				
None	67716	68.9	122316	67.6
Boy preference	15793	16.8	34153	18.8
Girl preference	13489	14.3	24564	13.6
**Age**				
15–19	4117	4.4	12588	7.0
20–24	14326	15.2	30986	17.1
25–29	20975	22.3	36494	20.2
30–34	18421	19.6	32060	17.7
35–39	15698	16.7	28153	15.5
40–44	11178	11.9	21434	11.8
45–49	9282	9.8	19317	10.7
**Educational level**				
No education	20529	21.8	83426	46.1
Primary	25724	27.4	69350	38.3
Secondary	38016	40.4	25568	14.1
Higher	9728	10.4	2688	1.5
**Employment status**				
Not working	23018	24.5	37348	20.6
Working	70980	75.5	143685	79.4
**Wealth index**				
Poorest	3703	3.9	51555	28.5
Poorer	6547	7.0	49630	27.4
Middle	13196	14.0	41547	22.9
Richer	26159	27.8	28586	15.8
Richest	44393	47.2	9715	5.4
**Religious affiliation**				
Christianity	61570	65.5	107365	59.3
Islam	29732	31.6	64273	35.5
Traditionalist	586	0.6	4302	2.4
No religion	2110	2.2	5093	2.8
**Intimate partner violence**				
No	76753	81.6	148406	82.0
Yes	17245	18.4	32627	18.0

**Table 3:** Prevalence of marital dissolution by place of residence across explanatory variables.

	Marital dissolution			
	Urban		Rural	
Variables	No	Yes	No	Yes
**Gender preference**				
None	89.46 [89.06–89.85]	10.54 [10.15–10.94]	92.65 [92.42–92.88]	7.35 [7.12–7.59]
Boy preference	91.18 [90.55–91.77]	8.82 [8.24–9.45]	94.05 [93.72–94.37]	5.95 [5.63–6.28]
Girl preference	88.74 [87-97-89.47]	11.26 [10.53–12.03]	91.93 [91.47–92.37]	8.07 [7.63–8.53]
**Age**				
15–19	91.12 [89.91–92.19]	8.88 [7.81–10.09]	93.10 [92.45–93.70]	6.90 [6.30–7.55]
20–24	90.32 [89.60–91.00]	9.68 [9.00–10.40]	92.26 [91.85–92.66]	7.74 [7.34–8.15]
25–29	90.29 [89.66–90.89]	9.71 [9.11–10.34]	93.43 [93.09–93.75]	6.57 [6.25–6.92]
30–34	89.28 [88.63–89.89]	10.72 [10.11–11.37]	93.28 [92.91–93.62]	6.72 [6.38–7.09]
35–39	89.18 [88.48–89.85]	10.82 [10.15–11.52]	92.83 [92.42–93.21]	7.17 [6.79–7.58]
40–44	88.65 [87.81–89.44]	11.35 [10.56–12.19]	92.52 [92.05–92.97]	7.48 [7.03–7.95]
45–49	89.24 [88.36–90.06]	10.76 [9.94–11.64]	91.93 [91.43–92.40]	8.07 [7.60–8.57]
**Educational level**				
No education	92.75 [92.20–93.26]	7.25 [6.74–7.80]	95.30 [95.08–95.52]	4.70 [4.48–4.92]
Primary	87.07 [86.44–87.66]	12.93 [12.34–13.56]	90.42 [90.10–90.73]	9.58 [9.27–9.90]
Secondary	89.12 [88.61–89.61]	10.88 [10.39–11.39]	91.16 [90.65–91.64]	8.85 [8.36–9.35]
Higher	91.79 [90.90–92.59]	8.22 [7.41–9.10]	92.92 [91.71–93.97]	7.08 [6.03–8.29]
**Employment status**				
Not working	92.34 [91.84–92.82]	7.66 [7.18–8.17]	93.75 [93.38–94.09]	6.25 [5.91–6.62]
Working	88.82 [88.41–89.21]	11.18 [10.79–11.59]	92.58 [92.35–92.80]	7.42 [7.20–7.65]
**Wealth index**				
Poorest	90.91 [89.31–92.29]	9.09 [7.71–10.69]	91.27 [90.88–91.64]	8.73 [8.36–9.12]
Poorer	90.92 [88.88–91.06]	9.98 [8.94–11.12]	93.39 [93.07–93.69]	6.61 [6.32–6.93]
Middle	89.57 [88.76–90.34]	10.43 [9.66–11.24]	93.31 [92.95–93.65]	6.69 [6.35–7.05]
Richer	88.87 [88.21–89.50]	11.13 [10.50–11.79]	93.70 [93.27–94.11]	6.30 [5.89–6.73]
Richest	89.98 [89.50–90.43]	10.02 [10.01–10.70]	93.40 [92.69–94.05]	6.60 [5.95–7.31]
**Religious affiliation**				
Christianity	87.96 [87.52–88.40]	12.04 [11.60–12.48]	90.72 [90.45–90.98]	9.28 [9.02–9.55]
Islam		6.97 [6.54–7.43]	95.99 [95.74–96.23]	4.01 [3.77–4.26]
Traditionalist	94.26 [91.78–96.02]	5.74 [3.98–8.22]	97.07 [96.34–97.66]	2.93 [2.34–3.66]
No religion	87.05 [84.92–88.91]	12.95 [11.09–15.08]	93.25 [92.19–94.18]	6.75 [5.82–7.81]
**Intimate partner violence**				
No	90.42 [90.06–90.76]	9.58 [9.24–9.94]	93.38 [93.17–93.58]	6.62 [6.42–6.83]
Yes	85.95 [89.30–89.99]	14.05 [10.01–10.70]	90.26 [89.82–90.67]	9.75 [9.33–10.18]

**Table 4:** Association between sex preference for children and marital dissolution in sub-Saharan Africa.

Variables	Null model	Model I	Model II	Model III	Model IV
		cOR (95% CI)	aOR (95% CI)	aOR (95% CI)	aOR (95% CI)
**Gender preference**					
None		Reference (1.0)	Reference (1.0)	Reference (1.0)	Reference (1.0)
Boy preference		0.80*** (0.77–0.83)	0.85*** (0.82–0.89)	0.84*** (0.81–0.87)	0.87*** (0.83–0.90)
Girl preference		1.11*** (1.07–1.15)	1.06** (1.02–1.11)	1.09*** (1.05–1.13)	1.06** (1.02–1.10)
**Age**					
15–19			Reference (1.0)		Reference (1.0)
20–24			1.05 (0.99–1.13)		1.02 (0.95–1.09)
25–29			1.00 (0.94–1.07)		0.96 (0.90–1.02)
30–34			1.09* (1.02–1.16)		1.02 (0.95–1.92)
35–39			1.12** (1.05–1.20)		1.06 (0.99–1.13)
40–44			1.17*** (1.09–1.26)		1.10** (1.03–1.18)
45–49			1.23*** (1.15–1.32)		1.20*** (1.12–1.29)
**Educational level**					
No education			Reference (1.0)		Reference (1.0)
Primary			1.73*** (1.64–1.79)		1.44*** (1.39–1.50)
Secondary			1.72*** (1.66–1.79)		1.42*** (1.36–1.49)
Higher			1.29*** (1.19–1.39)		0.99 (0.92–1.07)
**Employment status**					
Not working			Reference (1.0)		Reference (1.0)
Working			1.22*** (1.18–1.27)		1.37*** (1.32–1.41)
**Religious affiliation**					
Christianity			Reference (1.0)		Reference (1.0)
Islam			0.58*** (0.56–0.60)		0.66*** (0.64–0.69)
Traditionalist			0.37*** (0.32–0.43)		0.51*** (0.44–0.59)
No religion			0.96 (0.32–0.43)		1.05 (0.96–1.14)
**Intimate partner violence**					
No			Reference (1.0)		Reference (1.0)
Yes			1.41*** (1.36–1.46)		1.36*** (1.32–1.41)
**Wealth index**					
Poorest				Reference (1.0)	Reference (1.0)
Poorer				0.84*** (0.81–0.88)	0.78*** (0.75–0.81)
Middle				0.83*** (0.80–0.87)	0.76*** (0.73–0.80)
Richer				0.80*** (0.77–0.84)	0.74*** (0.70–0.77)
Richest				0.72*** (0.68–0.75)	0.68*** (0.65–0.72)
**Place of residence**					
Urban				Reference (1.0)	Reference (1.0)
Rural				0.54*** (0.52–0.56)	0.56*** (0.54–0.58)
**Subregion**					
Western Africa				Reference (1.0)	Reference (1.0)
Eastern Africa				2.39*** (2.30–2.48)	1.98*** (1.90–2.05)
Central Africa				1.74*** (1.67–1.82)	1.55*** (1.48–1.61)
Southern Africa				3.06*** (2.93–3.20)	2.17*** (2.07–2.28)
**Random effect results**					
PSU variance	0.03 (0.02–0.04)	0.03 (0.02–0.04)	0.04 (0.03–0.05)	0.03 (0.02–0.04)	0.03 (0.03–0.04)
ICC	0.0093477	0.0091651	0.0108115	0.0088723	0.0103676
Wald chi-square		195.25***	4105.16***	4439.25***	6336.04***
LR test	154.81 (X = 0.0000)	150.21 (X = 0.0000)	162.65 (X = 0.0000)	141.43 (X = 0.0000)	160.35 (X = 0.0000)
Model fitness					
Log-likelihood	-79261.853	-79160.82	-76965.19	-76904.035	-75740.357
AIC	158527.7	158329.6	153966.4	153832.1	151522.7
N	275,031	275,031	275,031	275,031	275,031
Number of groups	1612	1612	1612	1612	1612

aOR = Adjusted Odds Ratios; cOR = Crude Odds Ratio; CI = Confidence Interval

* *p* < 0.05

** *p* < 0.01

*** *p* < 0.001; 1.0 = Reference category; PSU = Primary Sampling Unit; ICC = Intra-Class Correlation; AIC = Akaike’s Information Criterion; LR test = Likelihood Ratio Test

Null Model is the Model with no explanatory variables or covariates

Model I had the outcome variable and the key explanatory variable

Model II had the outcome variable, key explanatory variable, and individual level covariates

Model III had the outcome variable, key explanatory variable, and community level covariates

Model IV had the outcome variable, key explanatory variable, and all the covariates

**Table 5:** Association between sex preference for children and marital dissolution by rural-urban strata in sub-Saharan Africa.

Variables	Marital dissolution	
	Urban	Rural
**Gender preference**		
	aOR (95% CI)	aOR (95% CI)
None	Reference (1.0)	Reference (1.0)
Boy preference	0.87** (0.80–0.95)	0.87*** (0.82–0.92)
Girl preference	1.06 (0.97–1.15)	1.07 (1.00–1.14)
**Age**		
15–19	Reference (1.0)	Reference (1.0)
20–24	0.96 (0.82–1.13)	1.02 (0.92–1.14)
25–29	0.96 (0.82–1.12)	0.89* (0.80–0.99)
30–34	1.06 (0.91–1.23)	0.91 (0.81–1.02)
35–39	1.08 (0.92–1.27)	1.01 (0.90–1.13)
40–44	1.14 (0.97–1.34)	1.05 (0.94–1.19)
45–49	1.12 (0.94–1.33)	1.22*** (1.08–1.37)
**Education**		
No education	Reference (1.0)	Reference (1.0)
Primary	1.32*** (1.20–1.45)	1.45*** (1.36–1.54)
Secondary	1.16** (1.05–1.28)	1.67*** (1.54–1.81)
Higher	0.82** (0.70–0.95)	1.3**(1.12–165)
**Employment status**		
Not working	Reference (1.0)	Reference (1.0)
Working	1.57*** (1.45–1.70)	1.21*** (1.13–1.29)
**Religious affiliation**		
Christianity	Reference (1.0)	Reference (1.0)
Islam	0.74*** (0.68–0.80)	0.62*** (0.58–0.67)
Traditionalist	0.59** (0.41–0.87)	0.50*** (0.39–0.64)
No religion	1.23* (1.02–1.47)	0.97 (0.83–1.14)
**Intimate partner violence**		
No	Reference (1.0)	Reference (1.0)
Yes	1.44*** (1.33–1.55)	1.39*** (1.32–1.47)
**Wealth index**		
Poorest	Reference (1.0)	Reference (1.0)
Poorer	1.07 (0.88–1.31)	0.69*** (0.65–0.74)
Middle	1.14 (0.95–1.38)	0.66*** (0.61–0.70)
Richer	1.21 (1.00–1.46)	0.58*** (0.53–0.63)
Richest	1.08 (0.89–1.30)	0.54*** (0.48–0.61)
**Sub-region**		
Western Africa	Reference (1.0)	Reference (1.0)
Eastern Africa	1.88*** (1.69–2.09)	2.21*** (2.02–2.43)
Central Africa	1.53*** (1.38–1.68)	1.57*** (1.41–1.74)
Southern Africa	2.31*** (2.06–2.58)	2.36*** (2.11–2.63)

aOR = Adjusted Odds Ratios; CI = Confidence Interval

* *p* < 0.05

** *p* < 0.01

*** *p* < 0.001; 1.0 = Reference category

### Ethical consideration

In this study, ethical clearance was not sought due to the public availability of the DHS dataset. However, we sought permission from the Monitoring and Evaluation to Assess and Use Results Demographic and Health Survey (MEASURE DHS) to use the datasets.

## Results

### Prevalence of marital dissolution among women in sub-Saharan Africa

**Fig 1:**
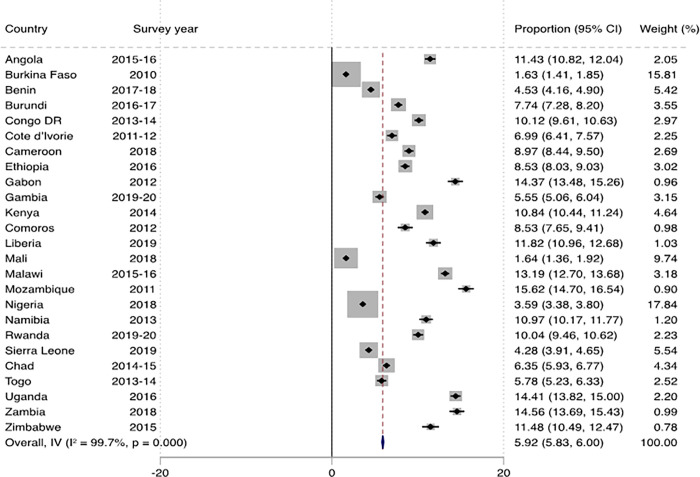
Prevalence of marital dissolution among women in sub-Saharan Africa.

**Fig 2:**
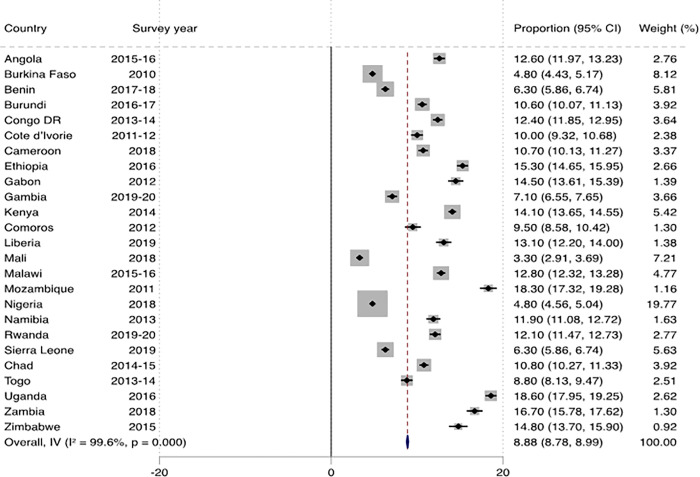
Prevalence of marital dissolution among women in urban sub-Saharan Africa.

**Fig 3:**
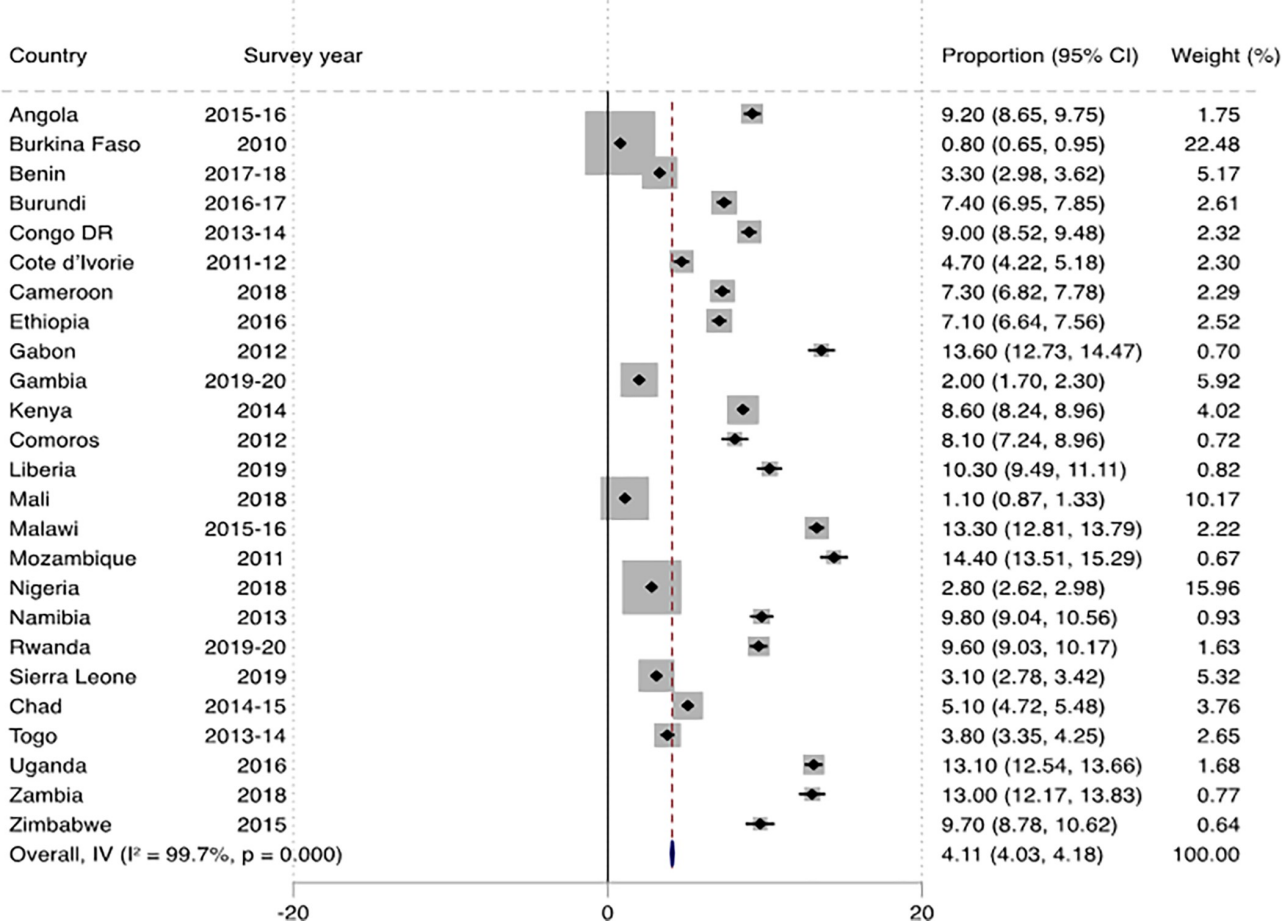
Prevalence of marital dissolution among women in rural sub-Saharan Africa.

Fig 1 shows the prevalence of marriage dissolution among women across countries in SSA. The overall prevalence of marital dissolution was 5.92% (95% CI: 5.83–6.00), and this ranged from 1.63% (95% CI: 1.41–1.85) in Burkina Faso to 15.62% (95% CI: 14.70–16.54) in Mozambique. In urban SSA, the overall prevalence of marital dissolution was 8.88% (95% CI: 8.78–8.99), with the lowest prevalence in Mali (3.30%; 95% CI: 2.91–3.69) and the highest in Uganda (18.60%; 95% CI: 17.95–19.25) (Fig 2). In rural SSA, the overall prevalence was 4.11% (95% CI: 4.03–4.18), with the lowest (0.80%; 95% CI: 0.65–0.95) and highest (14.40%; 95% CI: 13.51–15.29) prevalences in Burkina Faso and Mozambique respectively (Fig 3).

### Background characteristics of women in sub-Saharan Africa

Table 2 presents the results of the background characteristics of the respondents segregated by place of residence. It was found that in both rural (68.9%) and urban (67.6%) areas, the majority had no preference for sex of a child. With age, 22.3% in the urban and 20.2% of those in the rural were aged 25–29 years. With education, 40.4% of those in urban had secondary education while 46.1% of those in rural areas had no formal education. In terms of working status, 75.5% of those in urban and 79.4% of those in rural areas are working. For household wealth status, it was found that 47.2% of those in urban are in the richest wealth quintile whereas 28.5% of those in the rural areas are in the poorest wealth quintile. In both rural (59.3%) and urban (65.5%) areas, the majority are Christians.

### Prevalence of marital dissolution by place of residence across explanatory variables

The prevalence of marital dissolution by place of residence across explanatory variables is shown in Table 3. It was found that in both rural [8.07% [7.63–8.53] and urban (11.26% [10.53–12.03] areas, those with girl preference had the highest prevalence of experiencing marital dissolution. With age,11.35% [10.56–12.19] of those aged 40–44 in urban areas and 8.07% [7.60–8.57] of those aged 45–49 in rural areas had the highest prevalence of marital dissolution. With education and employment, those with primary education (12.93% in urban and 9.58% in rural) and those working (11.18% urban and 7.42% rural) had the highest prevalence of marital dissolution. It was also found that 11.13% of those in richer wealth quintile in urban areas and 8.73% of those in poorest wealth quintile had the highest prevalence of marital dissolution. With religious affiliation marital dissolution was predominant among Christians in both rural 9.28% and urban 12.04% areas as well as those who experience intimate partner violence (14.05% Urban areas and 9.75% in rural areas).

### Association between sex preference for children and marital dissolution among women in sub-Saharan Africa

Tables 4 and 5 show the results of the association between sex preference for children and marital dissolution by place of residence among women in SSA. Compared to women with no sex preference, the preference for boys was less likely to result in marital dissolution (aOR = 0.87; 95%CI = 0.83–0.90) in both urban and rural areas, whereas the preference for girls was more likely to result in marital dissolution (aOR = 1.06; 95%CI = 1.02–1.10). When the results were disaggregated by place of residence, in both urban (aOR = 0.87; 95%CI = 0.80–0.95) and rural areas (aOR = 0.87; 95%CI = 0.82–0.92), women who preferred boys were less likely to experience marital dissolution compared to those who had no preference. The covariates that showed significant associations with marital dissolution in both urban and rural areas were educational level, employment status, religion, and intimate partner violence. Women’s age and wealth index were significant covariates only in rural areas (Table 4, Model IV). When the results were disaggregated by place of residence, in both urban (aOR = 0.87; 95%CI = 0.80–0.95) and rural areas (aOR = 0.87; 95%CI = 0.82–0.92), women who preferred boys were less likely to experience marital dissolution compared to those who had no preference. The preference for girls was not associated with marital dissolution in both rural and urban areas (Table 5).

## Discussion

The present study examined the association between sex preference for children and marital dissolution across the dimension of place of residence. Overall, there is a low prevalence of marital dissolution across the 25 sub-Saharan African countries. From the results, it is indicative that in both rural and urban areas in SSA, male sex preference was significantly associated with a lower likelihood of marital dissolution. This agrees with a previous study conducted in Nigeria that found the risk of marital dissolution to be low among those who had preferred male children compared to those who preferred female children [6]. The probable mechanism or factors that underline this observation may include the influence of cultural norms and the values placed on male children. For instance, Roam and Railay [21] have asserted that children are wanted for dynastic imperative reasons. That is families, desire to pass on their name and ensure the continuity of their lineage. However, in the sub-Saharan African context, it is believed that the female child will lose the family name when they eventually get married. Hence, this cultural belief reinforces the dissolution of marriage in the event of the inability of women to bear male children who will perpetuate the family lineage [6]. The belief that the male child is a sort of economic security and a form of insurance against the unexpected could be another plausible reason for the low likelihood of marital dissolution among families with male child sex preference [22]. Likewise, the ‘father involvement hypothesis’ men are more involved in socialising male children than female children; thus, reinforcing interdependence between partners and making marital dissolution less probable in families that have male child preferences [16]. We also acknowledge that the association between male child preferences and marital dissolution may stem from the point that divorce and separation are less likely to occur in patriarchal settings. That is, the belief in the indissolubility of marriage, which is prevalent in patriarchal societies, can act as a deterrent to divorce or separation [23]. The societal emphasis on the preservation of the marital union may discourage individuals from pursuing divorce, even in the face of dissatisfaction or conflicts within the marriage. Such associations have been found for other regions such as historical Europe [24].

In both rural and urban areas, women who had ever experienced intimate partner violence were more likely to experience marital dissolution than those who had never experienced intimate partner violence. This result is similar to the findings from previous studies conducted in Nigeria [6] and SSA [25]. For instance, Seidu et al. [25] reported that women who experienced physical, emotional, and sexual violence from an intimate partner were more likely to have their marital union disrupted, thereby leading to marital dissolution. This result could be that women who experience intimate partner violence are likely to become less tolerant of violent behaviours and more probable to dissolve their marital union to escape further acts of violence. Another school of thought is that women who experience intimate partner violence are more likely to experience marital dissolution when the violence threatens their health and overall wellbeing [6].

Wealth status was only significant in predicting the likelihood of marital dissolution among rural-dwelling women. The study suggests an inverse association between wealth status and marital dissolution; that is, the odds of experiencing marital dissolution significantly reduce with increasing wealth status. Similar findings have been reported in SSA [25]. Higher wealth status among women may connote a higher sense of economic empowerment and autonomy. Hence, women of higher wealth status are less likely dependent on their partners. Consequently, they are more likely to easily end their marital unions when they are abused, or when they feel threatened or intimidated.

Relatedly, the study revealed that in both rural and urban areas, women who were employed had a higher likelihood of experiencing marital dissolution. The result is, however, incongruent with earlier findings that found an inverse association between employment status and odds of marital dissolution [26]. Nevertheless, the observed results could be explained from the perspective that being employed as a woman reduces the dependence on one’s partner thereby empowering them to dissolve marital unions that violates their rights [25]. Women who are employed may prioritise their progression on the job and self-actualisation rather than working to stabilise their marriage.

The study also showed that the likelihood of experiencing marital dissolution increased significantly with age. Our result is similar to a related study that found the odds of marital dissolution to be high among older women [6, 27]. However, this was only significant in urban areas. Possibly, this could be explained from the perspective that the fondness males have for women while they are younger may begin to wane as the women grow older [6].

Belonging to the traditionalist religion was associated with significantly low odds of marital dissolution. This observation is inconsistent with previous studies [6, 25] that found no significant association between religion and martial dissolution. Perhaps, the observed association between religion and marital dissolution could be due to the point that the traditional African religion is often grounded in patriarchal norms and systems that prefers male children and place much emphasis on male children.

### Strengths and limitations

Our study used a large, nationally representative dataset that has been validated. This supports the generalisability of the study findings. Also, appropriate statistical methods were employed in the conduct of the study. Notwithstanding, the cross-sectional nature of the DHS does not support the establishment of causality between sex preferences and marital dissolution. Also, sex preference for children and marital status were self-reported; hence, there is the likelihood of recall bias and social desirability bias. Finally, sex preference for children might be heavily dependent on the current set of offspring.

### Conclusion

This study has shown that sex preference for children has a significant association with marital dissolution in both rural and urban areas in SSA. The preference for male children serves as a protective factor against marital dissolution. Thus, underscoring a need for anti-marital dissolution campaigns and initiatives to prioritise the sensitisation of society about the value of female children. Religious groups and leaders can leverage their platform to quell sex preferences and dissuade marital dissolution. Policies and programmes formulated and implemented by government agencies (e.g., Department of Social Welfare) and non-governmental organisations to reduce the risk of marital dissolution in SSA must focus on enlightening the population on intimate partner violence prevention.

## Abbreviations

AIC: Akaike’s Information Criterion
aOR: Adjusted Odds Ratio
CI: Confidence Interval
cOR: Crude Odds Ratio
DHS: Demographic and Health Survey
ICC: Intra-Class Correlation
LR: Test: Likelihood-Ratio Test
PSU: Primary Sampling Unit
SSA: Sub-Saharan Africa
STROBE: Strengthening the Reporting of Observational Studies in Epidemiology
